# Dietary Knowledge, Dietary Adherence, and BMI of Lebanese Adolescents and Their Parents

**DOI:** 10.3390/nu12082398

**Published:** 2020-08-11

**Authors:** Liliane Said, Jessica S. Gubbels, Stef P. J. Kremers

**Affiliations:** 1 Department of Health Promotion, NUTRIM School of Nutrition and Translational Research in Metabolism, Faculty of Health, Medicine, and Life Sciences, Maastricht University, 6200 MD Maastricht, The Netherlands; jessica.gubbels@maastrichtuniversity.nl (J.S.G.); s.kremers@maastrichtuniversity.nl (S.P.J.K.); 2 Department of Nutrition and Food Sciences, Faculty of Arts and Sciences, Lebanese International University, Bekaa, Lebanon

**Keywords:** BMI, dietary knowledge, dietary adherence, nutrition, eating behaviour, adolescents, parents, Lebanon

## Abstract

Paediatric obesity is a severe public health problem accompanied by several physical and mental complications, mainly due to an imbalance between energy input and output. Dietary behaviours are influenced by many demographic factors and determinants, such as the place of residence and the level of dietary knowledge of the children and their parents. The aim of the current paper is to assess the levels of dietary knowledge, dietary adherence (in relation to recommendations), and the body mass index (BMI) of Lebanese adolescents in association with demographic variables, their parents’ dietary knowledge and adherence levels, and with other lifestyle behaviours. This cross-sectional study included 1535 Lebanese adolescents aged 15 to 18 years, from 16 public and private high schools located in urban and rural regions, and 317 of their parents. Our results showed that 30.2% of the adolescents were overweight or obese. Participants enrolled in private schools and those living in urban regions had a significantly higher BMI *z*-score compared to those enrolled in public schools and living in rural regions, respectively. In addition, Lebanese adolescents generally had low levels of dietary knowledge and 32.4% had low levels of dietary adherence. Their dietary adherence was significantly associated with their parents’ dietary adherence. The findings underline the significant role of the parents in shaping their children’s eating behaviours, in addition to the other determinants and factors affecting the diet of Lebanese adolescents. As the prevalence of paediatric overweight and obesity has reached alarming rates, the results of the current study have important implications for both public health policies and obesity prevention interventions in the Middle East and worldwide.

## 1. Introduction

Adolescence is a critical period for growth and development [1]. Among the various health problems faced by this age category is paediatric obesity [2]. According to the World Health Organisation (WHO), the prevalence of obesity among children and adolescents aged 5–19 years in Lebanon was 13.9% in 2016 [3]. Overweight and obesity can lead to numerous physical and mental complications [4]. Overweight and obesity are caused by an energy imbalance between the energy input (i.e., food intake) and the energy output (i.e., resting metabolic rate and physical activity), and are highly influenced by the obesogenic environment [5]. Among Lebanese adolescents specifically, the intake of sugar-sweetened beverages was found to be positively correlated with overweight, and higher intakes of milk and dairy products were negatively associated with overweight [6].

Many demographic factors affect eating behaviours, including the age, gender, degree of urbanisation, place of residence, and socio-economic status [7]. Studies have shown that older children had a significantly higher intake of energy-dense snacks compared to younger participants [8]. Furthermore, adolescent girls are more adherent to red meat intake guidelines compared to boys, but the intake of fruits and vegetables is low for both genders [9]. In Lebanon, obesity is more prevalent among women living in urban areas with a higher socio-economic status [10]. In addition, dietary knowledge plays an important role [7]: individuals with higher dietary knowledge levels are almost 25 times more likely to consume adequate amounts of fruits, vegetables, and fat compared to those with a lower level of dietary knowledge [11]. In addition, the clustering or co-occurrence of behaviours is another important factor influencing eating habits [12]. For instance, eating unhealthy snacks and watching television are two separate behaviours that are often performed simultaneously [12]. It seems that unhealthy behaviours often cluster with other unhealthy ones, and that healthy behaviours also often co-occur with other healthy behaviours. Other determinants of adolescents’ dietary intake include parental nutrition knowledge and food habits [7]. Romanos-Nanclares, et al. [13] reported that parental dietary knowledge of the recommended daily servings of food groups was positively correlated with the adequate consumption of dairy products, fruits, vegetables, and other food groups by their children. Similarly, healthier parental eating attitudes were associated with a greater consumption of fruits and vegetables, and a lower consumption of butter and meat by their children [13].

In Lebanon, studies have investigated neither the correlation between the dietary knowledge and dietary adherence of adolescents and children, nor the influence of the parents on their children’s dietary knowledge and adherence. In addition, very few studies have analysed the correlation between dietary habits and anthropometric outcomes of Lebanese adolescents. Moreover, there are no studies examining potential determinants related to the eating habits of Lebanese adolescents. Therefore, the aim of the current paper is to assess the levels of dietary knowledge, dietary adherence, and the BMI of Lebanese adolescents, as well as their association with demographic variables, parental dietary knowledge and adherence levels, and with clustered behaviours. This cross-sectional study included participants living in both urban and rural areas in Lebanon from different socio-economic backgrounds.

## 2. Materials and Methods

### 2.1. Study Design and Participants

The current cross-sectional study was conducted between October and December 2017, among 15- to 18-year-old adolescents attending public and private high schools in Beirut, Baalbeck, and Rayak in Lebanon, as well as their parents. Beirut, the capital of Lebanon, is an urban region, whereas Baalbeck and Rayak are rural regions. A total of 16 high schools were randomly selected from a list of high schools from the Ministry of Education, located in the selected locations: eight high schools from Beirut (six public and two private), six high schools from Baalbeck (three public and three private), and two high schools from Rayak (one public and one private). Participation was voluntary. Inclusion criteria for the participating adolescents included: (1) being Lebanese and enrolled in a Lebanese public or private high school located in one of the selected regions; (2) aged 15–18 years; (3) being fully capable (cognitive, psychiatric, and physical ability) of communicating (as reported by parents or by school administration); (4) not having any chronic or genetic diseases (as reported by parents or by school administration).

The required sample size (*n*) was directly proportional to the prevalence of overweight and obesity among Lebanese adolescents. As the adolescents participating in the previous pilot study [14] were highly cooperative, we did not oversample our required sample size to anticipate attrition [15]. This resulted in a minimally required sample size of 331 and 165, based on overweight and obesity prevalence, respectively.

The sampling method was nonselective, meaning that all participants meeting the inclusion criteria and present at the moment of data collection were approached. After receiving the consent of the school principal, all subjects gave their informed consent for inclusion before participating in the study. The questionnaire stated clearly that once it was filled in and handed in, this meant that the adolescent accepted and assented to participate in the study. A similar assent statement was also included in the parents’ version of the questionnaires. In addition, students’ consent was also orally confirmed by the principal investigator (PI) before administering the questionnaire. The study was conducted in accordance with the Declaration of Helsinki, and the protocol was approved by the Lebanese Ministry of Education and Higher Education (15,465/3/2016; date: 06/10/2017) and the Institutional Review Board of the Lebanese International University (LIUIRB-171,212-LS1). The final sample included 1535 adolescents and 317 parents (participation rate 19.4%). None of the approached adolescents refused to participate.

### 2.2. Data Collection

Data collection at schools was performed by a dietitian (PI) and a trained research assistant. Quality control measures, such as training, pre-testing of the study questionnaires, and data collection and data entry monitoring (e.g., data curation, double entry, range checks for data values, etc.), were applied. Data were collected using Arabic questionnaires (the native language of the participants). We describe the collected data in more detail below.

#### 2.2.1. Socio-Demographic Data

The socio-demographic information included the following: (1) age of the participants and their date of birth; (2) their gender; (3) the school class; (4) the type of school (public or private); and (5) the address (urban or rural).

#### 2.2.2. Dietary Knowledge

A Dietary Knowledge Questionnaire (DKQ) was administrated to adolescents and their parents. This questionnaire aims to assess the level of dietary knowledge of Lebanese adolescents and their parents. It was specifically designed for the current population and it was previously validated on a smaller similar sample of Lebanese adolescents (*N* = 220) and parents (*N* = 108) [14]. The DKQ consists of five parts, covering different nutrition-related themes [14]. After filling in the questionnaire, a total knowledge score is calculated by adding the points obtained on each item. The maximum possible score is 56, reflecting an extremely high level of dietary knowledge, and the minimum score is zero. The questionnaire showed an acceptable internal reliability, as Cronbach’s alpha was 0.82 for adolescents and 0.83 for the parents [14].

#### 2.2.3. Dietary Adherence

A Dietary Adherence Questionnaire (DAQ) was administrated to adolescents and their parents, aiming to assess their level of adherence to the dietary recommendations listed in the consensus statement from the American Heart Association [16] and the Dietary Reference Intakes [17]. This DAQ was designed specifically to suit the Lebanese population and it was previously pre-tested [14]. It consists of four parts. The total dietary adherence index is obtained by dividing the score for healthy items by the score for unhealthy items. If the resulting index is higher than 1, this means that the healthy food choices and habits outweigh the unhealthy ones. The opposite is true if the index is lower than 1. The maximum possible score is 37, reflecting a total adherence to dietary recommendations, and the minimum score is zero. The internal reliability was acceptable, as Cronbach’s alpha was 0.64 for adolescents’ healthy items and 0.61 for adolescents’ unhealthy items [14]. As for the parents, Cronbach’s alpha was 0.56 for both healthy and unhealthy items.

#### 2.2.4. Anthropometric Measurements

Weight and height were measured using standardised protocols and procedures [18] and calibrated equipment. Adolescents were weighed to the nearest 0.1 kg in light indoor clothing and without shoes [18]. Height was measured without shoes and recorded to the nearest 0.1 cm. Adolescents stood with their heels together, arms by their sides, legs straight, shoulders relaxed, and head in the Frankfort horizontal plane [18]. Body mass index (BMI) was calculated by dividing weight (kg) by height squared (m^2^) [18].

BMI is recognised as the most appropriate measure to detect paediatric obesity [19] and it is a strong predictor for total fat mass in children and adolescents older than nine years [20]. BMI measurement presents many advantages: it is inexpensive, relatively easy to obtain, non-invasive, and quick [21]. The WHO recommends using *z*-scores for consistency and comparability, compared to percentiles [22]. BMI *z*-scores, also known as BMI standard deviation (SD) scores, are measures of relative weight adjusted for age and gender [23]. In the current study, the gender- and age-specific BMI *z*-scores were calculated using the WHO AnthroPlus software [24]. As a frame of reference, BMI *z*-scores were classified as follows: ≤−3 indicating severe malnutrition; −2 to −2.9 indicating moderate malnutrition; −1 to −1.99 indicating mild malnutrition [25]; −0.99 to 1.03 indicating normal weight; 1.04 to 1.63 indicating overweight; 1.64 to 2.32 indicating obesity; and ≥2.33 indicating severe obesity [26].

Anthropometric measurements were obtained for 1418 out of 1535 (92.4%) adolescents. The rest of the participants were either absent during the measurement collection, refused to take their weight and/or height (e.g., because they were shy), or refused to follow the required procedure (e.g., refused to take their shoes off).

### 2.3. Statistical Analyses

Data were entered and analysed using the Statistical Package for Social Sciences version 25.0 (SPSS Inc., Chicago, IL, USA). We used descriptive statistics to analyse the participants’ characteristics, the average scores, the BMI *z*-score average, and the participants’ answers to the questionnaires. Independent *t*-tests were employed to determine significant differences between subgroup means (boys vs. girls, urban vs. rural, and public vs. private). Pearson’s correlations were used to explore potential relations between knowledge, the adherence scores of adolescents and parents, and BMI *z*-scores. Effect sizes were categorised to three groups: small (r = 0.10–0.29), medium (r = 0.30–0.49), and large (r ≥ 0.50) [27]. One-way analysis of variance (ANOVA) with a post hoc Tukey’s test was used to determine any differences between the three study locations (Beirut, Baalbeck, and Rayak). A *p* value of less than 0.05 was considered statistically significant. The obtained scores are expressed as mean and standard deviation (SD).

## 3. Results

### 3.1. Demographic Characteristics

The demographic characteristics of the participating adolescents and parents are shown in Table 1. The sample included more females than males (66.1% of the adolescents and 76.4% of their parents), more participants living in rural regions (67.3% of the adolescents and 50.2% of their parents), and more participants enrolled in public high schools (71.3% of adolescents and 90.5% of their parents).

### 3.2. Dietary Knowledge Score

Adolescents scored an average of 26.91 (SD = 7.46) out of 56 on the DKQ, and 59.2% answered more than half of the questions incorrectly. In terms of demographic characteristics, there was no significant difference in the knowledge score between groups, except for the location: adolescents from rural schools scored higher than those from urban ones (*p* < 0.001; see Table 2).

Parents scored an average total knowledge score of 35.29 (SD = 7.65) out of 56 on the DKQ, and there were no significant differences between the groups based on demographics, except for the type of school, with the parents of students from private schools scoring significantly higher (see Table 2). Detailed responses on the DKQ for both adolescents and parents are shown in the Supplementary Files—Table S1.

### 3.3. Dietary Adherence Index

The mean total dietary adherence index score was 1.80 (SD = 1.57), and 32.4% of adolescents obtained a score below 1 (i.e., healthy items score was lower than unhealthy items score). Girls scored significantly higher than boys (*p* = 0.02), and adolescents living in rural regions scored higher than adolescents living in urban regions (*p* < 0.001).

The mean healthy item score among adolescents was 8.83 (SD = 3.64) out of 37. There were significant differences between groups regarding gender, grade, and location. The mean unhealthy items score was 6.37 (SD = 3.19) out of 38, and there were significant differences between groups related to gender, type of school, and location (see Supplementary Files—Table S3).

In terms of physical activity, younger adolescents from grade 10 and from public schools tended to practice physical activity (PA) at school more than adolescents from grade 11 and from private schools (*p* < 0.001 for both). Similarly, adolescents living in urban regions tended to participate more in physical activity classes compared to adolescents from rural regions (*p* < 0.001). As for the screen-viewing time of adolescents, girls and adolescents living in urban regions tended to spend more time watching television and looking at smartphones, tablets, and other screens, compared to boys and adolescents living in rural regions (*p* < 0.001 and *p* = 0.011, respectively, see Supplementary Files—Table S4).

Parents had a mean total dietary adherence index of 2.69 (SD = 1.82), and there were no significant differences between the groups based on demographics (see Table 2).

The mean healthy items score among parents was 10.21 (SD = 3.31), and there were no significant differences between groups in terms of demographics except for gender. The mean unhealthy items score among parents was 4.87 (SD = 2.59), and there were no significant differences between groups, except for location (see Supplementary Files—Table S3). Detailed responses on the DAQ for both adolescents and their parents are shown in Supplementary Files—Table S2.

### 3.4. BMI z-Score

The mean BMI *z*-score of the adolescents was 0.44 (SD ± 1.20), ranging from −3.78 to 4.11 (see Table 3). Overall, more than half of the adolescents (59%) had a normal BMI *z*-score (−0.99 to 1.03). A total of 13.9% were overweight, 16.3% were obese, and 10.7% were underweight.

### 3.5. Correlations

#### 3.5.1. Knowledge and Adherence Scores

Table 4 shows the correlations between knowledge and adherences scores. There was a significant positive medium correlation between the total knowledge score of the adolescents and the total knowledge score of the parents (*p* < 0.001). There were significant small positive correlations between the total knowledge score of the adolescents and their healthy items (*p* < 0.001), and their total dietary adherence index (*p* < 0.001). In addition, a significant small negative correlation was found between the total knowledge score of adolescents and their unhealthy items score (*p* < 0.001).

The total knowledge score of the parents was significantly positively correlated with their total dietary adherence index (*p* = 0.007), and negatively correlated with their unhealthy items score (*p* = 0.01). The healthy items score of the parents was significantly positively correlated with the healthy items score of their children (*p* < 0.001), and the same medium positive correlation was found between the unhealthy items score of the parents and their children. In addition, the total dietary adherence indexes of the parents and adolescents were significantly positively correlated (*p* = 0.007). Detailed correlations between the DAQ items of the parents and adolescents are shown in Supplementary Files—Table S6.

#### 3.5.2. BMI *z*-Score and Total Knowledge and Adherence Scores

The BMI *z*-score of the adolescents was significantly positively correlated with their total knowledge score (*p* = 0.001) and the total knowledge score of the parents (*p* = 0.029). By contrast, the BMI *z*-score was significantly negatively correlated with the healthy items and unhealthy items score of adolescents (*p* < 0.01), and the healthy items score of the parents (*p* = 0.005; see Table 5). Detailed correlations between the BMI *z*-score and the DAQ items of adolescents are shown in Supplementary Files—Table S6.

#### 3.5.3. Clustered Behaviours

There was a significant negative correlation between the breakfast intake among adolescents and the intake of high-fat meats (*r* = −0.09; *p* = 0.001), the intake of commercial fruit juices (*r* = −0.08; *p* = 0.002), the number of snacks (*r* = −0.20; *p* < 0.001), and the screen-viewing time (*r* = −0.08; *p* = 0.004).

The physical activity of adolescents at schools was significantly and positively correlated with the following: the intake of vegetables (*r* = 0.06; *p* = 0.03), physical activity practice outside school (*r* = 0.25; *p* < 0.001), and the weekly number of hours spent on physical activity (*r* = 0.53; *p* < 0.001). There was a significant negative correlation with the intake of high-fat dairy products (*r* = −0.05; *p* = 0.049), the intake of commercial juices (*r* = −0.06; *p* = 0.03), the number of snacks (*r* = −0.07; *p* = 0.009), and screen-viewing time (*r* = 0.07; *p* = 0.012; see Supplementary Files—Table S5).

## 4. Discussion

To the best of our knowledge, the current study is the first to examine the dietary knowledge and dietary adherence levels of Lebanese adolescents from urban and rural regions and their parents, as well as the correlation between them and their association with the BMI *z*-scores of adolescents.

Adolescents scored relatively low on the knowledge score. A previous study on 9- to 11-year-old Lebanese children showed higher knowledge scores (average 63% compared to 48% in the current study). The interviewers of the current study insisted that if participants did not know the answer to a question, they should indicate “do not know” rather than provide random answers. As this eliminates filling in the correct answer by chance, this might have influenced the lower knowledge score. However, another cross-sectional study reported that 86% of the participating United Arab Emirati adolescents had a low level of dietary knowledge [28]. With regard to the parents, their average dietary knowledge score was 63%, thus scoring higher than their children. This is in line with another study reporting similar knowledge scores among parents [29].

The average total adherence index among adolescents was above 1, meaning that, overall, the healthy items outweighed unhealthy ones, in line with a previous study [14]. These findings are in line with previous studies in various countries. In a study using the Healthy Eating Index, Brazilian adolescents scored an average of 51.8 (out of 100) [30]. In European countries, poor compliance with the dietary recommendations was observed in 37.6% of children [31], in line with the 32.4% found in the current study. Additionally, the current study showed relatively low healthy items scores, compared to another study among adolescents aged 15–18 years from New Zealand using the Healthy Dietary Habits for adolescents index [32].

With regard to the parents, the total dietary adherence index was well above 1, and the healthy items score was also higher than their children’s scores. It therefore seems that parents’ diets are healthier than those of their adolescent children. To our knowledge, there are no studies assessing the level of dietary adherence of parents specifically, but a study in Qatar among adults in general reported similar dietary adherence scores [33]. Another study including Saudi adults showed a lower level of dietary adherence (average score of 47.4%) [34].

Overall, the present study showed high rates of overweight and obesity among Lebanese adolescents (13.9% and 16.3%, respectively). In particular, the percentage of obesity appeared high. This could be due to the nutrition transition affecting Lebanese children and adolescents who are adopting the Western lifestyle, characterised by long hours of screen-viewing time and the excessive consumption of fast food [6]. In a previous study among Lebanese youth, the prevalence of overweight (30.8%) was higher [6], and the prevalence of obesity was lower (10.3%). Another cross-sectional study including Jordanian adolescents also reported higher rates of overweight (15.7%) and lower rates of obesity (8.7%) [35], although different cut-off points were used in both studies to interpret the BMI *z*-scores. Furthermore, the BMI *z*-score was significantly higher among boys compared to girls in the current study, in line with other studies reporting that boys are at a higher risk for obesity [6]. As for the type of school, the average BMI *z*-score was significantly higher in private high schools compared to public ones. Thus, adolescents with a higher socio-economic status (more often attending private schools with higher tuition fees [36]), tend to be overweight or obese more often. Similar to many developing countries, in Lebanon, obesity is more prevalent among individuals with a higher socio-economic status [10]. Furthermore, the average BMI *z*-score was also higher among urban participants compared to rural individuals, which is also reported in other studies conducted in Lebanon and Jordan [6,35].

However, underweight is another nutritional problem that this age category faces, with 10.7% of the current adolescents being undernourished. In a study in Tunisia, the prevalence of underweight among a similar sample of adolescents was lower (4.8%) [37]. Underweight among adolescents was reported in many Arab countries, ranging from 5 to 25% [10]. These numbers indicate the presence of both types of malnutrition (under- and overnutrition) in one country and among a critical age category, requiring further attention. This is assumed to be mainly due to unhealthy eating habits and a lack of dietary knowledge [10]. The double burden of a high prevalence of both overweight and underweight in many developing countries has devastating public health and economic consequences, especially in low-income countries [38].

Despite mostly small effect sizes, the significant positive correlation between the dietary knowledge of adolescents and the total dietary adherence index shows that knowledge is one of the factors influencing the dietary habits of Lebanese adolescents. In other words, the more adolescents know about nutrition, the better they follow the dietary guidelines. These findings are in line with other studies reporting that university students with a higher nutrition knowledge score were more likely to follow the dietary guidelines [39]. The current results also showed that the level of the parents’ dietary knowledge influences their children’s eating behaviours only indirectly, by influencing their children’s dietary knowledge; a direct correlation between parents’ knowledge and children’s eating was not evident. Zarnowiecki, Sinn, Petkov, and Dollman [29] also reported that dietary knowledge levels of children and their parents were positively correlated. Studies among younger children (8–11 years) do report a direct correlation between parents’ knowledge and their children’s healthy food intake [40], which might reflect the gatekeeping role parents have for younger children’s eating behaviour [41]. In adolescents, parents have less control over what their children eat [42]. Nonetheless, adolescents’ eating behaviours were similar to their parents’ dietary habits. Parents frame family diets, shape the development of culturally appropriate eating patterns in children, and are role models for eating behaviour [41].

As for the parents, we noticed a positive correlation between their total knowledge score and the adherence index, reflecting once again that knowledge is an important determinant of dietary habits [7]. There was also a negative correlation between the unhealthy items score and the knowledge score, but there was no significant correlation between the healthy items score and the knowledge level. This means that, among parents, the dietary knowledge may act as an inhibitor of unhealthy eating habits rather than as a stimulator of healthy eating habits. These individuals may thus be aware of what should be avoided, but not necessarily of the available healthy alternatives. A similar correlation between the knowledge and the healthy and unhealthy items scores was found in a previous study among Lebanese adolescents [14].

A positive correlation between the BMI *z*-score of adolescents and their dietary knowledge score was found, which might indicate that overweight and obese children tend to be more interested in nutrition compared to other children. Similarly, parents of children with higher BMI *z*-scores tended to be more knowledgeable about nutrition as well. In line with this, Milosavljević, et al. [43] reported that the minimum knowledge score of adolescents was higher among those with a higher BMI. However, a study conducted in Jordan found that mothers with a high to moderate level of knowledge were more likely to have a child with a decreased waist circumference, but their knowledge was unrelated to their children’s BMI [44]. Other available studies did not measure the BMI *z*-score, but rather only the BMI, failing to adjust for gender and age differences in BMI among adolescents [21]. The BMI *z*-score of the current adolescents was further significantly negatively correlated with the intake of both the healthy items and the unhealthy items. Previous research has also shown that adolescents with higher BMIs tend to adopt less healthy behaviours [45]. However, their lower consumption of unhealthy items indicates that overweight and obese adolescents tend to eat less food overall (both healthy and unhealthy), perhaps to decrease their weight. Alternatively, there might be a higher social desirability bias among overweight and obese adolescents. In addition, the negative correlation between the BMI *z*-score and the healthy items score of the parents indicates that the healthy eating habits and PA practices of the parents influence the weight of their children. This is because parents following healthy habits may influence their children to adopt similar healthier choices (see correlation of healthy items score of adolescents and their parents), thereby leading to a decreased BMI *z*-score. To our knowledge, studies have not evaluated the association between the child’s BMI *z*-score and the healthy and unhealthy behaviours of the parents separately.

The main strength of the present study is its large and diverse sample covering both urban and rural regions and different types of high schools. Data collection was performed according to standardised and quality-controlled procedures. This is the first study in the region to investigate the correlation between the dietary knowledge and dietary adherence of adolescents and their parents. It is also the first study to examine the correlation between the BMI *z*-score of Lebanese adolescents and the levels of dietary knowledge and dietary adherence of adolescents and their parents. The current study allows us to understand the dietary behaviour of Lebanese adolescents and the factors influencing it. In addition, all the questionnaires used were pre-tested and designed specifically to suit the Lebanese population [14]. Furthermore, the study focused on both healthy and unhealthy food and health-related behaviours.

Some limitations should be also considered. First, the sample did not include participants from all the Lebanese regions, and it is therefore unclear whether the results can be generalised to the larger population. Second, the sample was not equally distributed over the different subgroups (e.g., based on gender, region). These differences in the sample subgroups were expected based on previous studies conducted on a similar sample, with more girls than boys, as the percentage of girls enrolled in Lebanese high schools is higher compared to boys [14]. Similarly, the number of participants from rural regions was higher than those from urban regions, despite having included fewer rural schools, because the number of students per class in rural schools is higher compared to urban high schools [14]. In line with this, an unequal number of participants between public and private schools could have been expected [14]. This was also observed in other studies outside Lebanon [35,46]. Third, the dietary knowledge and adherence variables were based on self-reports rather than on objective assessment, potentially causing bias. However, direct measurements (e.g., observation) are not feasible in studies with a large population [31].

### Implication for Research, Practice, and Policy

Our findings are of interest to researchers, health promoters, educators, dietitians, and policy makers involved in improving the nutritional status and diet quality of adolescents worldwide. This study is the first step preceding the implementation of a dietary intervention to ameliorate the eating habits of Lebanese adolescents. Such interventions should be planned carefully to suit individuals with different BMI *z*-scores to prevent further weight loss among adolescents with undernutrition (10.7%). Health promoters should target the factors with the highest effect sizes with regard to BMI *z*-score and dietary adherence to make interventions more effective and more efficient, especially knowing that schools have limited time when it comes to extra-curricular activities. In addition, interventions should focus on individuals with the lowest dietary knowledge and adherence scores, e.g., adolescents from urban regions. Additionally, as some healthy behaviours co-occur with other healthy eating habits, future interventions must emphasise the importance of these specific behaviours (e.g., breakfast consumption and decreased screen-viewing time) to enhance their effectiveness.

## 5. Conclusions

This study indicates that the prevalence of overweight and obesity has reached alarming rates among Lebanese adolescents and it emphasises the importance of implementing interventions to prevent paediatric obesity among the youth population. Such interventions should target behaviours that are associated with a lower BMI *z*-score, such as breakfast consumption, and focus on nutrition topics for which adolescents show the lowest knowledge and dietary adherence. National studies to investigate the change in obesity rates, as well modifications in the dietary knowledge and adherence levels in Lebanon, are recommended.

## Figures and Tables

**Table 1:** Demographic characteristics of the participants.

	Adolescents (*N* = 1535)	Parents (*N* = 317)
Age, mean (SD)	15.8 (0.8)	43.3 (8.2)
Gender, *n* (%)		
Male	520 (33.9)	72 (23.6)
Female	1015 (66.1)	233 (76.4)
Location, *n* (%)		
Beirut (U)	501 (32.6)	158 (49.8)
Baalbeck (R)	834 (54.3)	122 (38.5)
Rayak (R)	200 (13.0)	37 (11.7)
Type of school, *n* (%)		
Public	1094 (71.3)	287 (90.5)
Private	441 (28.7)	30 (9.5)
Grade, *n* (%)		
Grade 10	870 (57.0)	181 (57.3)
Grade 11	655 (43.0)	135 (42.7)

U: Urban; R: Rural; SD: Standard deviation.

**Table 2:** Means (SD) and differences in total knowledge and adherence scores among adolescents and their parents.

	Adolescents				Parents			
	Total Knowledge Score	*p* Value	Total Dietary Adherence Index	*p* Value	Total Knowledge Score	*p* Value	Total Dietary Adherence Index	*p* Value
Overall	26.91 (7.46)	-	1.80 (1.57)	-	35.29 (7.65)	-	2.69 (1.82)	-
Gender								
Male	26.45 (8.37)	0.10	1.68 (1.39)	0.02	34.21 (6.56)	0.11	2.86 (2.08)	0.40
Female	27.15 (6.94)		1.87 (1.65)		35.86 (7.84)		2.64 (1.73)	
Class								
Grade 10	26.60 (7.27)	0.10	1.80 (1.52)	0.86	34.62 (7.77)	0.07	2.81 (1.90)	0.16
Grade 11	27.24 (7.70)		1.82 (1.65)		36.20 (7.46)		2.50 (1.69)	
Type of school								
Public	26.70 (6.85)	0.11	1.83 (1.46)	0.40	34.87 (7.64)	<0.01	2.68 (1.71)	0.79
Private	27.44 (8.76)		1.75 (1.83)		39.37 (6.59)		2.78 (2.76)	
Location								
Urban	25.36 (7.80)	<0.01	1.61 (1.28)	<0.01	35.84 (7.33)	0.21	2.78 (1.63)	0.41
Rural	27.66 (7.17)		1.91 (1.70)		34.75 (7.94)		2.60 (1.99)	
Beirut (U)	25.36 (7.80)	<0.01	1.61 (1.28)	<0.01	35.84 (7.33)	0.27	2.78 (1.63)	0.33
Baalbeck (R)	27.59 (6.99)		1.87 (1.50)		34.42 (7.89)		2.70 (2.19)	
Rayak (R)	27.99 (7.86)		2.04 (2.30)		35.86 (8.14)		2.25 (0.99)	

Notes: Maximum points are 56 for dietary knowledge (DKQ) and >1 for dietary adherence (DAQ) reflecting better adherence to healthy rather than unhealthy behaviours. Differences were examined using an independent *t*-test for binary variables and ANOVA post hoc for differences between Beirut, Baalbeck, and Rayak. Abbreviations: DKQ: Dietary Knowledge Questionnaire; DAQ: Dietary Adherence Questionnaire; U: Urban; R: Rural.

**Table 3:** Means of BMI *z*-scores among Lebanese adolescents.

	Mean BMI *z*-Score (SD)
*Overall*	0.44 (1.20)
Gender **	
*Boys*	0.58 (1.33)
*Girls*	0.38 (1.12)
Grade	
*Grade 10*	0.43 (1.22)
*Grade 11*	0.45 (1.17)
Type of school **	
*Public*	0.38 (1.16)
*Private*	0.60 (1.28)
Location **	
*Urban*	0.57 (1.27)
*Rural*	0.38 (1.16)

Notes: ** Within each group, mean BMI *z*-scores are significantly different at *p* < 0.01. Differences were examined using an independent *t*-test. Abbreviations: BMI: Body mass index; SD: Standard deviation

**Table 4:** Correlations between knowledge and adherence scores of the adolescents and their parents.

		Adolescents			Parents			
		Healthy Items Score	Unhealthy Items Score	Total Dietary Adherence Index	Total Knowledge Score	Healthy Items Score	Unhealthy Items Score	Total Dietary Adherence Index
**Adolescents**	Total Knowledge score	0.12 ***	−0.13 ***	0.14 ***	0.26 ***	0.09	−0.04	0.10
	Healthy Items score		0.15 ***	0.45 ***	−0.02	0.28 ***	0.09	0.10
	Unhealthy Items score			−0.54 ***	−0.05	0.04	0.29 ***	−0.15 *
	Total Dietary Adherence index				0.08	0.15 *	−0.14 *	0.16 **
**Parents**	Total Knowledge score					0.11	−0.15 *	0.16 **
	Healthy Items score						0.27 ***	0.30 ***
	Unhealthy Items score							−0.62 ***

* *p* < 0.05; ** *p* < 0.01; *** *p* < 0.001 (Using Pearson’s correlation).

**Table 5:** Correlations between knowledge and adherence scores and BMI *z*-scores of adolescents.

	Total Knowledge Score of Adolescents	Total Dietary Adherence Index of Adolescents	Healthy Items Score of Adolescents	Unhealthy Items Score of Adolescents	Total Knowledge Score of Parents	Total Dietary Adherence Index of Parents	Healthy Items Score of Parents	Unhealthy Items Score of Parents
**BMI *z*-Score of Adolescents**	0.09 **	0.02	−0.11 ***	−0.09 **	0.13 *	−0.01	−0.17 **	−0.08

* *p* < 0.05; ** *p* < 0.01; *** *p* < 0.001 (Using Pearson’s correlation).

## Supplementary Materials

The following are available online at https://www.mdpi.com/2072-6643/12/8/2398/s1. Table S1: Responses to the Dietary Knowledge Questionnaire by the adolescents and their parents. Table S2. Responses to the Dietary Adherence Questionnaire of the adolescents and their parents. Table S3. Between group comparisons of the healthy items and unhealthy items scores of the adolescents and their parents. Table S4. Between group comparison of separate Dietary Adherence Questionnaire (DAQ) items according to demographic characteristics. Table S5: Pearson’s correlations between the DAQ items of adolescents. Table S6: Pearson’s correlations between the DAQ items of adolescents and the DAQ items of the parents, the total knowledge score of adolescents, and the BMI *z*-score.

## References

[1] National Academies of Sciences, Engineering, and Medicine (2019). The Promise of Adolescence: Realizing Opportunity for All Youth.

[2] World Health Organization (2016). Report of the Commission on Ending Childhood Obesity.

[3] WHO Prevalence of Obesity Among Children and Adolescents, BMI > +2 Standard Deviation above the Median, Crude Estimates by Country, among Children Aged 5–19 Years. http://apps.who.int/gho/data/node.main.BMIPLUS2C?lang=en.

[4] Yanovski J.A. (2015). Pediatric obesity. An introduction. Appetite.

[5] Alberga A.S., Sigal R.J., Goldfield G., Prud’homme D., Kenny G.P. (2012). Overweight and obese teenagers: Why is adolescence a critical period?. Pediatric Obes..

[6] Nasreddine L., Naja F., Akl C., Chamieh M.C., Karam S., Sibai A.-M., Hwalla N. (2014). Dietary, Lifestyle and Socio-Economic Correlates of Overweight, Obesity and Central Adiposity in Lebanese Children and Adolescents. Nutrients.

[7] Stok M., Hoffmann S., Volkert D., Boeing H., Ensenauer R., Stelmach-Mardas M., Kiesswetter E., Weber A., Rohm H., Lien N. (2017). The DONE framework: Creation, evaluation, and updating of an interdisciplinary, dynamic framework 2.0 of determinants of nutrition and eating. PLoS ONE.

[8] Gevers D.W., Kremers S.P., de Vries N.K., van Assema P. (2016). Intake of energy-dense snack foods and drinks among Dutch children aged 7–12 years: How many, how much, when, where and which?. Public Health Nutr..

[9] Batis C., Aburto T.C., Sanchez-Pimienta T.G., Pedraza L.S., Rivera J.A. (2016). Adherence to Dietary Recommendations for Food Group Intakes Is Low in the Mexican Population. J. Nutr..

[10] Musaiger A.O., Hassan A.S., Obeid O. (2011). The Paradox of Nutrition-Related Diseases in the Arab Countries: The Need for Action. Int. J. Environ. Res. Public Health.

[11] Wardle J., Parmenter K., Waller J. (2000). Nutrition knowledge and food intake. Appetite.

[12] Kremers S.P., de Bruijn G.-J., Visscher T.L., van Mechelen W., de Vries N., Brug J. (2006). Environmental influences on energy balance-related behaviors: A dual-process view. Int. J. Behav. Nutr. Phys. Act..

[13] Romanos-Nanclares A., Zazpe I., Santiago S., Marin L., Rico-Campa A., Martin-Calvo N. (2018). Influence of Parental Healthy-Eating Attitudes and Nutritional Knowledge on Nutritional Adequacy and Diet Quality among Preschoolers: The SENDO Project. Nutrients.

[14] Said L., Gubbels J.S., Kremers S.P.J. (2019). Development of Dietary Knowledge and Adherence Questionnaires for Lebanese Adolescents and Their Parents. Int. J. Environ. Res. Public Health.

[15] Bartlett J., Kotrlik J., Higgins C. (2001). Organizational Research: Determining Appropriate Sample Size in Survey Research. Inf. Technol. Learn. Perform. J..

[16] Gidding S.S., Dennison B.A., Birch L.L., Daniels S.R., Gilman M.W., Lichtenstein A.H., Rattay K.T., Steinberger J., Stettler N., Van Horn L. (2005). Dietary Recommendations for Children and Adolescents A Guide for Practitioners: Consensus Statement From the American Heart Association. Circulation.

[17] Institute of Medicine (2005). Dietary Reference Intakes for Water, Potassium, Sodium, Chloride, and Sulfate.

[18] Lee R., Nieman D. (2012). Nutritional Assessment.

[19] Stewart L., Shaw V. (2015). Obesity. Clinical Paediatric Dietetics.

[20] Vanderwall C., Randall Clark R., Eickhoff J., Carrel A.L. (2017). BMI is a poor predictor of adiposity in young overweight and obese children. BMC Pediatrics.

[21] Nihiser A.J., Lee S.M., Wechsler H., McKenna M., Odom E., Reinold C., Thompson D., Grummer-Strawn L. (2007). Body mass index measurement in schools. J. Sch. Health.

[22] Anderson L.N., Carsley S., Lebovic G., Borkhoff C.M., Maguire J.L., Parkin P.C., Birken C.S. (2017). Misclassification of child body mass index from cut-points defined by rounded percentiles instead of Z-scores. BMC Res. Notes.

[23] Must A., Anderson S.E. (2006). Body mass index in children and adolescents: Considerations for population-based applications. Int. J. Obes..

[24] De Onis M., Onyango A.W., Borghi E., Siyam A., Nishida C., Siekmann J. (2007). Development of a WHO growth reference for school-aged children and adolescents. Bull. World Health Organ..

[25] Becker P., Carney L.N., Corkins M.R., Monczka J., Smith E., Smith S.E., Spear B.A., White J.V. (2015). Consensus statement of the Academy of Nutrition and Dietetics/American Society for Parenteral and Enteral Nutrition: Indicators recommended for the identification and documentation of pediatric malnutrition (undernutrition). Nutr. Clin. Pract..

[26] Hoelscher D.M., Kirk S., Ritchie L., Cunningham-Sabo L. (2013). Position of the Academy of Nutrition and Dietetics: Interventions for the Prevention and Treatment of Pediatric Overweight and Obesity. J. Acad. Nutr. Diet..

[27] Field A. (2013). Discovering Statistics Using IBM SPSS Statistics.

[28] Al-Yateem N., Rossiter R. (2017). Nutritional knowledge and habits of adolescents aged 9 to 13 years in Sharjah, United Arab Emirates: A crosssectional study. East. Mediterr. Health J..

[29] Zarnowiecki D., Sinn N., Petkov J., Dollman J. (2012). Parental nutrition knowledge and attitudes as predictors of 5-6-year-old children’s healthy food knowledge. Public Health Nutr..

[30] Horta P.M., Verly Junior E., Santos L.C.D. (2019). Usual diet quality among 8- to 12-year-old Brazilian children. Cad. Saude Publica.

[31] Kovacs E., Siani A., Konstabel K., Hadjigeorgiou C., de Bourdeaudhuij I., Eiben G., Lissner L., Gwozdz W., Reisch L., Pala V. (2014). Adherence to the obesity-related lifestyle intervention targets in the IDEFICS study. Int. J. Obes..

[32] Wong J.E., Skidmore P.M., Williams S.M., Parnell W.R. (2014). Healthy dietary habits score as an indicator of diet quality in New Zealand adolescents. J. Nutr..

[33] Al Thani M., Al Thani A.A., Al-Chetachi W., Al Malki B., Khalifa S.A.H., Bakri A.H., Hwalla N., Naja F., Nasreddine L. (2018). Adherence to the Qatar dietary guidelines: A cross-sectional study of the gaps, determinants and association with cardiometabolic risk amongst adults. BMC Public Health.

[34] Alkhaldy A.A., Aljahdli E.S., Mosli M.H., Jawa H.A., Alsahafi M.A., Qari Y.A. (2020). Adherence to the Saudi dietary guidelines and its relation to colorectal polyps: A university hospital-based study. J. Taibah Univ. Med. Sci..

[35] Abu Baker N.N., Daradkeh S.M. (2020). Prevalence of overweight and obesity among adolescents in Irbid governorate, Jordan. East. Mediterr. Health J..

[36] Habib-Mourad C., Ghandour L.A., Moore H.J., Nabhani-Zeidan M., Adetayo K., Hwalla N., Summerbell C. (2014). Promoting healthy eating and physical activity among school children: Findings from Health-E-Pals, the first pilot intervention from Lebanon. BMC Public Health.

[37] Aounallah-Skhiri H., Romdhane H.B., Traissac P., Eymard-Duvernay S., Delpeuch F., Achour N., Maire B. (2008). Nutritional status of Tunisian adolescents: Associated gender, environmental and socio-economic factors. Public Health Nutr..

[38] Fernandez A., Martínez R. (2017). The Cost of the Double Burden of Malnutrition: Social and Economic Impact—Summary of the Pilot Study in Chile, Ecuador and Mexico.

[39] Kresic G., Kendel Jovanović G., Pavicic Žeželj S., Cvijanovic O., Ivezic G. (2009). The effect of nutrition knowledge on dietary intake among Croatian university students. Coll. Antropol..

[40] El-Nmer F., Salama A., Elhawary D. (2014). Nutritional knowledge, attitude, and practice of parents and its impact on growth of their children. Menoufia Med. J..

[41] Savage J.S., Fisher J.O., Birch L.L. (2007). Parental influence on eating behavior: Conception to adolescence. J. Law Med. Ethics.

[42] Story M., Neumark-Sztainer D., French S. (2002). Individual and Environmental Influences on Adolescent Eating Behaviors. J. Am. Diet. Assoc..

[43] Milosavljević D., Mandić M.L., Banjari I. (2015). Nutritional knowledge and dietary habits survey in high school population. Coll. Antropol..

[44] Subih H.S., Abu-Shquier Y., Bawadi H., Al-Bayyari N. (2018). Assessment of body weight, maternal dietary knowledge and lifestyle practices among children and adolescents in north Jordan. Public Health Nutr..

[45] Al Junaibi A., Abdulle A., Sabri S., Hag-Ali M., Nagelkerke N. (2013). The prevalence and potential determinants of obesity among school children and adolescents in Abu Dhabi, United Arab Emirates. Int. J. Obes..

[46] Maia E.G., Silva L., Santos M.A.S., Barufaldi L.A., Silva S.U.D., Claro R.M. (2018). Dietary patterns, sociodemographic and behavioral characteristics among Brazilian adolescents. Rev. Bras. Epidemiol..

